# Discussion of Follow-Up and Treatment Results of Children With High-Grade Renal Trauma

**DOI:** 10.7759/cureus.51618

**Published:** 2024-01-03

**Authors:** Nurcan Çoşkun, Adnan Abasiyanik

**Affiliations:** 1 Pediatric Surgery, Hitit University Erol Olçok Training and Research Hospital, Çorum, TUR; 2 Pediatric Surgery, Necmettin Erbakan University, Konya, TUR

**Keywords:** pediatrics, blunt abdominal injury, high-grade, differential renal function, renal trauma

## Abstract

Purpose: The aim of the study is to examine the follow-up and treatment results of late renal functions in children with high-grade (Grades 4, 5) renal trauma resulting from blunt abdominal injury.

Methods: The follow-up and treatment reports of 41 patients with renal trauma admitted to our clinic between the years 2005 and 2015 were reviewed retrospectively.

Results: Eight of the 41 cases had Grade 1, five had Grade 2, and 12 had Grade 3 renal trauma. The remaining 16 cases (12 of which were Grade 4, four were Grade 5) had high grade renal trauma. Four (25%) patients with high-grade renal trauma were operated (JJ stent placement was performed on one, renorraphy was performed on two, pyeloplasty and urinoma drainage were performed on one), and 12 patients were followed conservatively. In the long-term follow-up (>1 year), Tc-99m mercaptoacetyltriglycine (MAG3) kidney scintigraphy examination of three (30%) patients out of the 10 patients who were followed up conservatively, the affected kidneys were found to be nonfunctional (renal differential function 0%). The mean differential renal function in four patients who underwent surgery was 31% (between 25% and 40%).

Conclusion: It should be kept in mind that kidneys may become atrophic or non-functional in the late period of follow-up in cases that are followed conservatively due to high-grade renal trauma. There is no standard algorithm or treatment method in the management of high-grade renal trauma. In order to achieve a good outcome, the treatment should be individualized as much as possible.

## Introduction

Despite all the developments in modern medicine, half of child deaths occur due to trauma. Approximately 10% of these traumas impact the genitourinary system [1]. Kidney injuries are the most frequently encountered injuries among urinary system traumas, and forms approximately 50% of all urinary system injuries [2]. More than 90% of kidney injuries in the pediatric patients are due to blunt abdominal trauma [3]. Since the kidneys occupy a proportionally larger amount of space in the abdominal cavity during childhood, they are more vulnerable to trauma, accordingly kidney damage is more serious as compared to adults. The location of the kidneys in the abdomen is relatively lower, and the protective perirenal fat tissue and subcutaneous fat tissue are thinner in children than in adults. Furthermore, due to the immaturity of the rib cage in youngsters, it exhibits greater elasticity and receives less support from the surrounding muscles compared to adults. Due to its anatomical form, the renal pedicle is more susceptible to deceleration injuries in comparison to adults. Another factor contributing to the increased susceptibility of parenchyma to injury is the ongoing process of fetal lobulation in children [4].

The treatment of kidney injuries has long been one of the controversial topics among trauma surgeons. While a conservative treatment approach is adopted in low- and moderate-grade (Grades 1, 2, 3) kidney traumas, life-saving surgical interventions may be necessary in high grade (Grades 4, 5) injuries [5]. Today, a significant portion of high-grade injuries can be successfully treated without surgery in any hospital where close follow-up can be performed and with adequate medical equipment. Creating a treatment plan is the most difficult decision among the patients whose vital signs are stable despite having a high-grade kidney injury [6].

In this study, we aimed to compare operative and non-operative treatments with early and late functional results of blunt kidney injuries to determine which treatment is the most suitable for high-grade injuries.

## Materials and methods

After obtaining institutional review board committee approval (ID14567952-050), 41 patients diagnosed with kidney injury due to blunt abdominal trauma who were followed up and treated in the pediatric surgery department between January 2005 and December 2015 were evaluated, retrospectively.

Causes of trauma, additional organ injuries if any, the degree of kidney injury with IV contrast-enhanced abdominal tomography (CT) (Grades 1-5), hematuria status, blood transfusion amount, and hospitalization period of the patients were analyzed along with demographic characteristics such as age and sex, and the distribution of operatively and non-operatively treated cases [7].

The cases were divided into three groups as mild, moderate, or high degree according to the degree of kidney injury. Group 1 consisted of Grade 1 and 2 cases; Group 2 consisted of Grade 3 cases; and Group 3 consisted of Grade 4 and 5 cases.

During the follow-up of these 41 cases, 29 late (≥1 year) and 12 early (third month) MAG3 kidney scintigraphy were performed, the early and late-stage renal functions were determined, and the efficiency of operative and non-operative treatments were compared. In high-grade injuries, the criteria for operative and non-operative treatments were evaluated, and blood urea nitrogen (BUN), high creatinine, and development of hypertension were also analyzed.

Statistical analysis

All statistical analyses of the study were carried out with SPSS 19.0 package program. Descriptive measures of all variables obtained from the cases were calculated. Normality test of continuous numerical variables was carried out with Kolmogorov-Smirnov and Shapiro-Wilks tests. However, it was observed that the variables were not normally distributed (p<0.05). Therefore, for group comparisons, Mann-Whitney U analyses were used in the case of two independent groups and Kruskal-Wallis analyses in multiple groups. Wilcoxon analysis was used for the comparison of the repeated measurements. Monte Carlo or Fisher exact tests were used for the correction of chi-square test to examine the relationship between categorical variables. Pairwise comparisons were made in the multi-group analyses that were found to be significant. Type-I error level was accepted as 5% in all analyses, and a p value <0.05 value was considered as statistically significant.

## Results

The statistical findings regarding the data analysis were presented in Table 1.

**Table 1:** Findings by renal injury grades MDRF: Mean differential renal function; F: Female; M: Male; TA: Traffic accident; FFH: Fall from height; WA: Work accident; IKI: Isolated kidney injury; MI: Multiple injury

Grades	Mean Age	Gender		Cause of Trauma			Additional Organ Injury		Hematuria		Hospital Stay (Days)	Treatment Approach		MDRF
		F	M	TA	FFH	WA	IKI	MI	YES	NO		Conservative Treatment	Surgical Treatment	
Grade 1 (n=8)	9.6	4	4	4	4	-	4	4	2	6	9.5	8	-	48.17
Grade 2 (n=4)	7.7	2	2	3	1	-	1	3	0	4	5.75	4	-	47.23
Grade 3 (n=12)	9.6	5	7	9	3	-	3	9	4	8	11.25	12	-	41.44
Grade 4 (n=12)	9.6	4	7	8	3	-	7	4	6	6	11.09	10	2	28.46
Grade 5 (n=4)	10.25	1	3	2	1	1	1	3	2	2	12.25	2	2	25.07

Of the 41 cases, 43.9% (n=18) were female, and 56.1% (n=23) were male. The mean age of the patients was 10 years (1-17 years). Causes of injury were traffic accidents in 65.9% of the cases (n=27), falling from height in 31.7% of the cases (n=13), and work accidents in 2.4% of the cases (n=1). Of the cases examined, 39% (n=16) had kidney injuries isolated, and 61% (n=25) had added organ injuries. Kidney injuries were most frequently accompanied with spleen injuries with a rate of 31.7% (n=13).

Out of the 41 cases with kidney injuries in our study, eight had Grade 1 injury (19.5%), four had Grade 2 injury (9.7%), 12 had Grade 3 injury (29.2%), 12 had Grade 4 injury (29.2%), and four had Grade 5 injury (9.7%). The degree of injury could not be determined in one patient due to loss of abdominal CT medical records. Patients were divided into three groups according to the grade of their injury as mild, moderate, and severe kidney injury groups. Group 1 consisted of mild injuries with Grades 1 and 2 (12 patients, 29.5%); Group 2 consisted of moderate injuries with Grade 3 (12 patients, 29.5 %); and Group 3 consisted of severe injuries with Grades 4 and 5 (16 patients, 39 %).

The mean hospital stay was seven days in Group 1 (ranging from 3 to 24 days), 9.5 days (ranging from 5 to 33 days) in Group 2, and 10 days (ranging from 5 to 20 days) in Group 3. As the degree of injury increased, the length of hospital stay increased as well. However, this relationship was not statistically significant (p=0.141). Macroscopic hematuria was present in 16.7% of Group 1, 33.3% of Group 2, and 56.2% of Group 3. The incidence of macroscopic hematuria increased significantly as the degree of injury increased (p=0.033). Nine of 16 patients (56.2%) with isolated kidney injury required blood transfusion, and seven of these nine patients had hematuria. The need for blood transfusion was significantly higher in those with hematuria (p=0.042).

**Figure 1:**
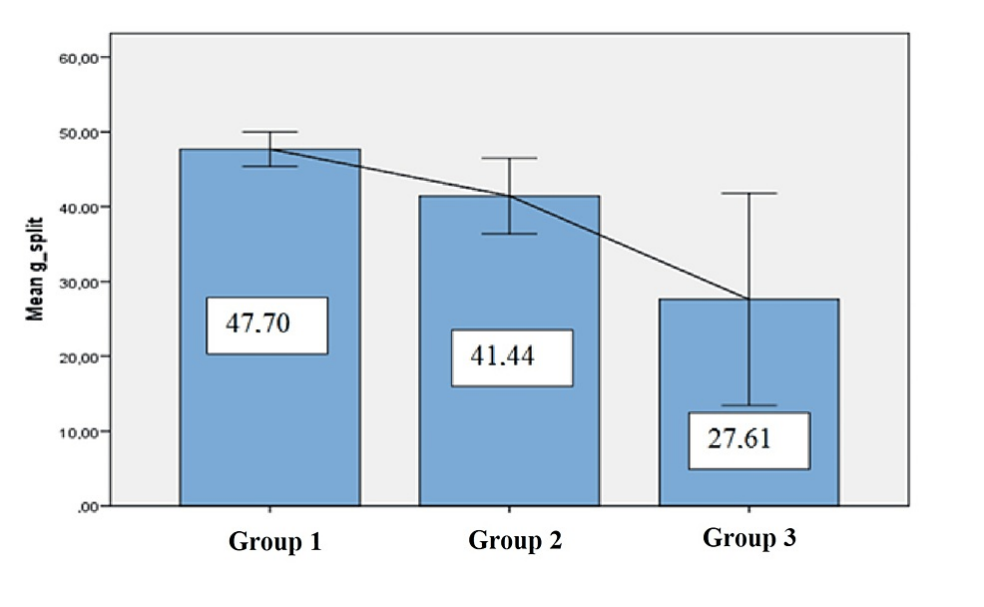
Split renal functions of the injured kidneys at Tc-99m MAG3 kidney scintigraphy according to groups MAG3: Mercaptoacetyltriglycine

The mean renal differential functions of the cases were 47.7%±2.3 in Group 1, 41.4%±14.1 in Group 2, and 27.6%±14.1 in Group 3. As the degree of injury increased, loss of function significantly increased, as well (p<0.001) (Figure 1).

**Figure 2:**
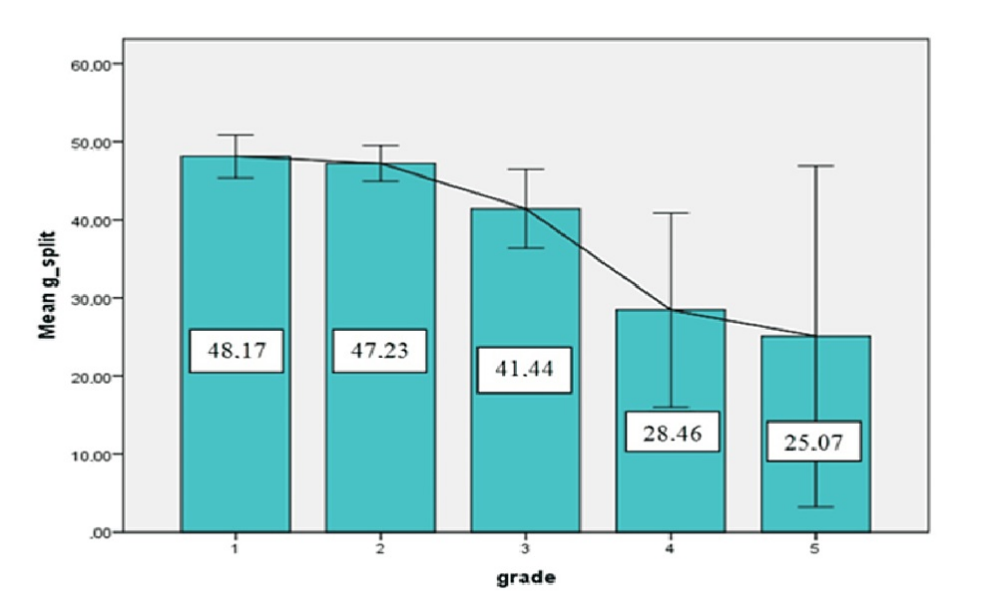
Split reanl functions of the injured kidneys at Tc-99m MAG3 kidney scintigraphy according to kidney injury grades (1-5) MAG3: Mercaptoacetyltriglycine

According to kidney injury grades, the mean differential function of cases with Grade 1 injuries was 48.1±2.7%, those with Grade 2 injuries was 47.2±2.2%, those with Grade 3 injuries was 41.4±5.05%, and those with Grade 4 injuries was 28.4%±12.4, and 25.0±21.8% for those with Grade V injuries. It was observed that as the grade increased, differential function losses increased significantly (p=0.002) (Figure 2).

While 90.3% (n=37) of the patients were followed conservatively, only four patients (9.7%) underwent surgical intervention. JJ stent placement was performed in one patient with Grade 4 injury and progressively increasing pyelectasis observed in follow-up examinations, and laparotomy was performed in two patients with Grade 5 injury and urine extravasation observed in IV contrast abdominal tomography. Pyeloplasty and urinoma drainage were performed in one patient who had a Grade 4 injury concurrent with ureteropelvic obstruction and had urinary in IV contrast abdominal tomography.

In Group 3 with high-grade renal injuries, the late-term renal differential functions of the patients who underwent surgical treatment and those who were followed up conservatively were compared. The renal differential functions of the patients who underwent surgical treatment were 31.16%±10.8, while the functions of those who were followed conservatively were 26.42%±15.5, and there was no statistically significant difference between the two groups (p=0.864). However, the kidneys of 3 of the patients who underwent conservative treatment were found to be dysfunctional (renal differential function 0%). In addition, early and late renal differential functions of each case were compared, and no statistically significant difference was found between them (p=0.769).

Out of these 41 cases in our study, 29 of whom were followed up in our center were also investigated for BUN, elevated creatinine, and hypertension. BUN and creatinine values were normal in all patients, including the 3 patients with dysfunctional kidneys, and all patients were normotensive.

## Discussion

Trauma is a preventable situation that falls under the broad responsibilities of pediatric surgery. Kidneys are the most frequently injured organ among urinary system traumas, and constitute approximately 50% of all urinary system injuries [2]. In kidney injuries, the primary goal of treatment is to preserve the kidney or the functioning kidney tissue.

The most important objective in creating the treatment plan is making a correct diagnosis. CT with IV contrast is the gold standard in the diagnosis and grading of blunt kidney injuries. Contrast-enhanced CT allows more detailed evaluation of the renal parenchyma, vascular structures, and collecting system as compared to ultrasonography (USG). In our retrospective study covering a 15-year period, we opted to use CT with IV contrast for the initial diagnosis and grading of the kidney damage, and subsequently we utilized USG for the follow-up of the patients [7]. In some studies evaluating the efficacy of USG in the diagnosis of renal trauma, it has been reported that moderate and high-grade kidney injuries can be detected with USG, but low-grade injuries may go unnoticed [7,8]. According to kidney injury grades, the mean differential function of cases with Grade 1 injuries was 48.1±2.7%, those with Grade II injuries was 47.2±2.2%, and those with Grade 3 injuries was 41.4±5.05%. Almost all low-grade renal injuries can be treated conservatively without significant loss of function. Although low-grade injuries may go unnoticed with USG, we think that these undetected injuries will not lead to complication or mortality, and therefore, USG is a reliable test for the rapid evaluation of blunt kidney injuries.

Macroscopic hematuria was observed in 16 of 41 cases in our study (39%). The results of multiple studies examining whether there is a correlation between hematuria and the severity of renal injury have shown that hematuria is an important but nonspecific finding. It has been reported in the literature that there is no correlation between the degree of microscopic hematuria and the degree of renal injury [9-11]. However, the incidence of macroscopic hematuria is higher in high-grade kidney injuries [12]. In our study, the incidence of macroscopic hematuria in patients with high-grade (Grades 4 and 5) renal injury was found to be significantly higher than those with moderate and mild injuries (Grades 1, 2 and 3) (p=0.033). In addition, the need for blood transfusion was found to be significantly higher in cases with macroscopic hematuria (p=0.042). Based on these data, we think that the kidneys should be screened in all cases with macroscopic hematuria, so that clinically significant injuries that require close follow-up and treatment of the patient are not overlooked. In addition, considering the low-grade probability of overlooked injuries, we believe that this situation will not affect the result.

It has been shown that 3-20% of kidneys injured as a result of trauma have renal anomalies [3]. In our study, horseshoe kidney was observed in one patient (2.4%), and ureteropelvic obstruction was observed in another patient (2.4%).

Conservative treatment has become the standard for low-grade blunt renal injuries [8]. However, there is no algorithm or standard treatment for serious injuries [13]. In high-grade kidney injuries, if the patient is hemodynamically stable, they are initially followed conservatively. When the literature is examined, the only surgical indication that has been agreed upon is hemodynamic instability. The patient group that has the most difficulty in treatment is the one in which patients are hemodynamically stable despite having high-grade injuries [7,8,14]. Today, it is seen that minimally invasive techniques significantly reduce the need for open surgery in this patient group [13]. Since two-thirds of urinoma resolve spontaneously, endourological methods are only used for symptomatic urinoma that grow over time or persist for a long time [13,15,16]. Angioembolization is the first line of treatment in hemodynamically stable patients with ongoing or delayed bleeding. When compared with surgical treatment, angioembolization has been reported to cause less morbidity, lower complication rates, faster recovery, and less kidney loss [17]. In cases where bleeding cannot be stopped and hemodynamic stability is impaired, the treatment option is surgery.

In the last 20 years, the conservative approach has become an important treatment option in blunt kidney injuries. A substantial amount of research supports conservative approach, even in high-grade renal injuries, based on the high rates of kidney salvage with this approach [15]. The goals of conservative treatment are avoiding unnecessary nephrectomy, limiting renal function loss, and treating complications such as urinary extravasation, infection, and bleeding. The rarity of complications and the ability of the kidney to heal are the main advantages of conservative treatment [18]. However, many studies do not discuss the short- and long-term functional results of kidneys preserved with conservative treatment [7,8,12,17,19-21].

Reports of successful conservative treatments, even for Grade 5 injuries, prompted the authors to investigate the functional consequences of this treatment. In some of these studies, post-traumatic renal function was evaluated only by serum creatinine levels, and in others by Tc-99m dimercaptosuccinic acid (DMSA) kidney scintigraphy performed three months and one year after trauma [8]. In all studies examining renal function according to serum BUN and creatinine levels, these values were found to be normal regardless of the treatment method [8,22,23]. In our study, the serum BUN creatinine level was found to be normal in all of the cases, which is in line with the literature. In addition, our patients were asymptomatic and normotensive in their follow-up, which is in accordance with the findings in the literature [22,24].

Some researchers also examined kidney functions with DMSA scintigraphy performed three months and one year after the trauma, and reported that the loss of renal function was directly proportional to the degree of injury. While good functional outcome was reported in all Grade 1, 2, and 3 injuries, it was noted that Grade 5 injuries resulted in significant loss of renal function regardless of the treatment method [23-27]. In our study, it is seen that the loss of kidney function increases as the degree of injury increases. In Groups 1 and 2, differential renal functions of only two (8.3%) patients with Grade 3 injuries were observed to be between 30-40%, while the functions of all the others were above 40%. These results support that conservative treatment is an effective treatment method in mild and moderate kidney injuries. Low-grade injuries do not require long-term follow-up as they involve fewer surgical procedures and a low risk of complications, but high-grade injuries require long-term follow-up as they involve greater functional loss [24].

In two studies evaluating Grade 3, 4, and 5 injuries, it was reported that kidney functions were preserved (between 40-50%) [22,28]. However, in these studies, Grade 3 injuries were also included in the group with serious injuries [23].

In addition, it has been reported in the literature that early and late functional outcomes are similar in kidneys affected by trauma [23]. The similarity of early and late-term results in our study shows that late-term results can be predicted by looking at early-term functional results.

There is limited research comparing the efficacy of operative and conservative treatment. While a study reported no difference in mean reduction in function when controlled for grade between those treated operatively and conservatively, another study reported that those who were treated conservatively had better functions than those who required surgical intervention [18,25]. In our study, it was seen that the mean of the differential functions of those who were treated operatively was higher than those who were treated conservatively, but this difference was statistically insignificant. It was found that three (30%) of the conservatively treated patients developed dysfunctional kidneys in their long-term follow-up.

Complications such as persistent bleeding, progressive urinoma formation, fever and infection are more common in Grade 5 renal traumas (fragmented kidney or major vascular injury). There is usually severe loss of renal function. Operative treatment and nephrectomy required more often [2,14,24]. In recent years, successful results have been reported with conservative treatment in Grade 5 renal trauma. However, based on the data in our study, we think that it should be kept in mind that non-functional kidneys can be seen after conservative treatment. Therefore, deciding on operative or conservative treatment is the most difficult and the most important part of the treatment. There is no standard algorithm or treatment method for high-grade cases. The approach should be as individualized as possible to achieve a good result. Our study has limitations such as being retrospective and having a small number of Group 3 cases. Further prospective studies including higher number of patients may provide more significant results.

## Conclusions

It should be kept in mind that atrophic or non-functioning kidneys may occur at a substantial level in the late period in high-grade renal traumas and in cases treated conservatively. We believe that surgical treatment is necessary in patients whose bleeding cannot be stopped, who are hemodynamically unstable, and/or who have a ruptured kidney. Although rare, complications such as increased BUN and creatinine levels, as well as hypertension, may occur. Therefore, it is important to monitor patients by doing kidney function tests and measuring blood pressure regularly. The absence of a significant difference between early and late term functions indicates that late renal functions can be predicted by looking at early renal functions. Also, there is no standard algorithm or treatment method for high-grade cases. In order to achieve a good outcome, the treatment should be individualized as much as possible.
